# Transthyretin deposition promotes progression of osteoarthritis

**DOI:** 10.1111/acel.12665

**Published:** 2017-09-22

**Authors:** Tokio Matsuzaki, Yukio Akasaki, Merissa Olmer, Oscar Alvarez‐Garcia, Natalia Reixach, Joel N. Buxbaum, Martin K. Lotz

**Affiliations:** ^1^ Department of Molecular Medicine The Scripps Research Institute La Jolla CA USA

**Keywords:** aging, amyloid, cartilage, osteoarthritis, transthyretin

## Abstract

Deposition of amyloid is a common aging‐associated phenomenon in several aging‐related diseases. Osteoarthritis (OA) is the most prevalent joint disease, and aging is its major risk factor. Transthyretin (TTR) is an amyloidogenic protein that is deposited in aging and OA‐affected human cartilage and promotes inflammatory and catabolic responses in cultured chondrocytes. Here, we investigated the role of TTR *in vivo* using transgenic mice overexpressing wild‐type human *TTR* (hTTR‐TG). Although TTR protein was detected in cartilage in hTTR‐TG mice, the *TTR* transgene was highly overexpressed in liver, but not in chondrocytes. OA was surgically induced by destabilizing the medial meniscus (DMM) in hTTR‐TG mice, wild‐type mice of the same strain (WT), and mice lacking endogenous *Ttr* genes. In the DMM model, both cartilage and synovitis histological scores were significantly increased in hTTR‐TG mice. Further, spontaneous degradation and OA‐like changes in cartilage and synovium developed in 18‐month‐old hTTR mice. Expression of cartilage catabolic *(Adamts4, Mmp13)* and inflammatory genes (*Nos2, Il6*) was significantly elevated in cartilage from 6‐month‐old hTTR‐TG mice compared with WT mice as was the level of phospho‐NF‐κB p65. Intra‐articular injection of aggregated TTR in WT mice increased synovitis and significantly increased expression of inflammatory genes in synovium. These findings are the first to show that TTR deposition increases disease severity in the murine DMM and aging model of OA.

## Introduction

Osteoarthritis (OA) is the most prevalent human joint disease with age being the main risk factor (Zhang & Jordan, 2010; Losina *et al*., 2013). There are currently no established approaches to prevent or slow the progression of OA (Zhang & Jordan, 2010) and over half of adults with knee OA undergo knee replacement (Weinstein *et al*., 2013). A large number of therapeutic targets have been identified and successfully tested in animal models (Yu & Hunter, 2015). However, thus far clinical trials targeting these pathways have failed (Hunter & Hellio Le Graverand‐Gastineau, 2009). Changes in articular cartilage appear to be the earliest event in disease initiation and are likely to be the main drivers of disease progression, but all the joint tissues are affected by the disease process (Loeser *et al*., 2012). Age‐related changes in cartilage have been characterized, but the mechanisms that mediate the effect of age on OA are unknown.

In studies of articular cartilage, menisci, and synovium from arthritic joints, the prevalence of amyloid deposits was between thirty and one hundred percent of joints examined (Goffin, 1983; Ladefoged, 1986; Solomon *et al*., 2006; Gobezie *et al*., 2007; Niggemeyer *et al*., 2011). Amyloid deposits in OA synovium were ranged from 8% (Takanashi 2013) to 25% (Gu *et al*. 2014) of the patients. The most common precursor is the thyroxin (T4) and retinol transporter protein transthyretin (TTR) (Goffin, 1983). TTR is mainly produced by hepatocytes, choroid plexus epithelium, and retinal pigment epithelium. It is found in blood plasma, the cerebrospinal fluid, and the vitreous humor of the eye. The protein is composed of four identical subunits (Blake *et al*., 1978). TTR amyloid formation requires tetramer dissociation into monomers that misfold and aggregate to initiate the amyloidosis cascade (Hurshman *et al*., 2004). In contrast with the less common forms of autosomal‐dominant familial amyloidotic polyneuropathy (FAP) and cardiomyopathy (FAC), the more prevalent senile systemic amyloidosis (SSA) is caused by the deposition of amyloid derived from wild‐type TTR (Buxbaum *et al*., 2008). It occurs mainly in elderly males, with its clinically dominant manifestations related to deposits of wild‐type TTR in the myocardium (Rapezzi *et al*., 2013). It may be accompanied by carpal tunnel syndrome with TTR deposition in the carpal ligament or gastrointestinal bleeding due to the involvement of blood vessels (Sekijima *et al*., 2011).

We have previously reported that all human cartilage samples collected at the time of joint replacement surgery were positive for amyloid by Congo red staining and TTR by immunohistochemistry and Western blotting (Akasaki *et al*., 2015). In addition, we showed in studies of primary cultured chondrocytes that exposure to amyloidogenic TTR affected chondrocyte survival and induced the expression of OA‐related genes (Akasaki *et al*., 2015). These findings raise the question of whether the deposits contribute to the process of cartilage degradation. It is also possible that the damaged tissues create an environment, which supports TTR tetramer dissociation, misfolding of the released monomers, and subsequent aggregation to oligomers and fibrils, which in turn could amplify the OA process. The objective of this study was to investigate the role of TTR *in vivo* in mice transgenic for 90‐100 copies of the wild‐type human TTR (hTTR‐TG mice) using an experimental OA and aging model.

## Results

### Pattern of TTR deposition in mouse knee joints and sources of TTR

As hTTR‐TG mice had not previously been examined for developmental changes in the skeleton, analysis of whole skeleton preparations of newborn (P2) mice was performed. They did not reveal any apparent developmental abnormalities and the long bone lengths were not significantly different from WT mice (Fig. S1).

As previously reported, Western blotting showed strong signals for human TTR in serum and liver from 9‐month‐old hTTR‐TG mice (Teng *et al*., 2001). In articular cartilage from 9‐month‐old hTTR‐TG mice, TTR was present at a much lower concentration than in serum or liver. However, no TTR protein was detected in the chondrocytes of these mice (Fig. S2A). There was no TTR in the cartilage, chondrocytes, liver, or serum from mTTR‐KO mice. PCR analysis showed a detectable but very low level of hTTR mRNA in cartilage from hTTR‐TG mice compared to liver (Fig. S2B). In 2‐month‐old WT mice, there were similarly low levels of mTTR mRNA in joint tissues compared to liver (Fig. S2C). These results indicate that the TTR protein found in cartilage and synovium comes from systemic rather than local sources.

**Figure 1:**
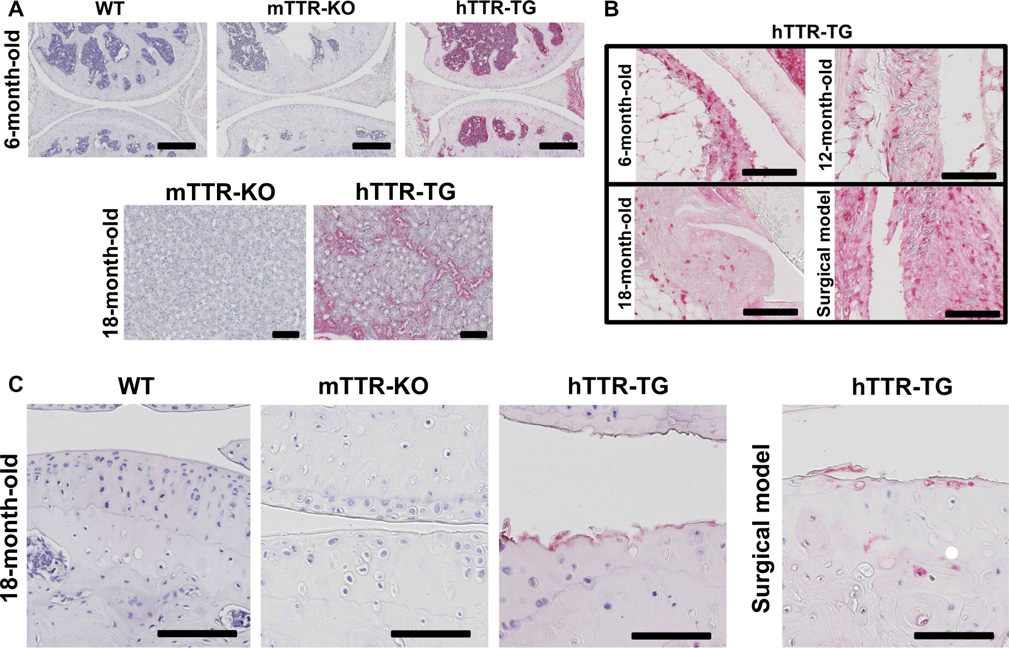
Immunohistochemistry for transthyretin (TTR) in bone marrow, kidney, and synovium. (A) TTR was detected in bone marrow and blood vessels of 6‐month‐old hTTR‐TG but not in wild‐type (WT) and mTTR‐KO mice, and around blood vessels in the kidney of 18‐month‐old hTTR‐TG mice (upper: scale bars = 400 μm, lower: scale bars = 100 μm). (B) TTR was detected in the synovium of 6‐, 12‐, and 18‐month‐old hTTR‐TG mice and hTTR‐TG mice with surgical OA (scale bars = 100 μm). (C) TTR was detected in fibrillated cartilage in 18‐month‐old hTTR‐TG mice and hTTR‐TG mice with surgical OA (scale bars = 100 μm). WT and mTTR‐KO mice showed no TTR staining.

Immunohistochemical analysis revealed TTR deposits in bone marrow and around blood vessels in the kidney of hTTR‐TG mice (Fig. 1A). TTR deposits were also evident in the synovium of 6‐, 12‐, and 18‐month‐old mice and 6‐month‐old mice with surgically induced OA (Fig. 1B). However, TTR deposition in cartilage was not detected in 6 (*n* = 5)‐ and 12‐month‐old (*n* = 14) hTTR‐TG mice, whereas 69% of the 18‐month‐old mice (9/13), and 100% of the mice (5/5) with surgically induced OA had TTR staining in cartilage. The predominant areas of TTR deposition were the regions of fibrillated cartilage in the 18‐month‐old hTTR‐TG mice and in the younger mice subjected to destabilization of the medial meniscus (Fig. 1C). The antibody used in Fig. 1 recognizes human but not mouse TTR. With an antibody that recognizes TTR in human and mouse, TTR was detected in bone marrow and blood vessels in 6‐month‐old hTTR and WT mice and at the articular cartilage surface in 18‐month‐old hTTR‐TG mice but not in WT and mTTR‐KO mice (Fig. S5).

### hTTR‐TG mice show severe cartilage damage, synovitis and subchondral bone changes in an experimental OA model and in the aging model

**Figure 2:**
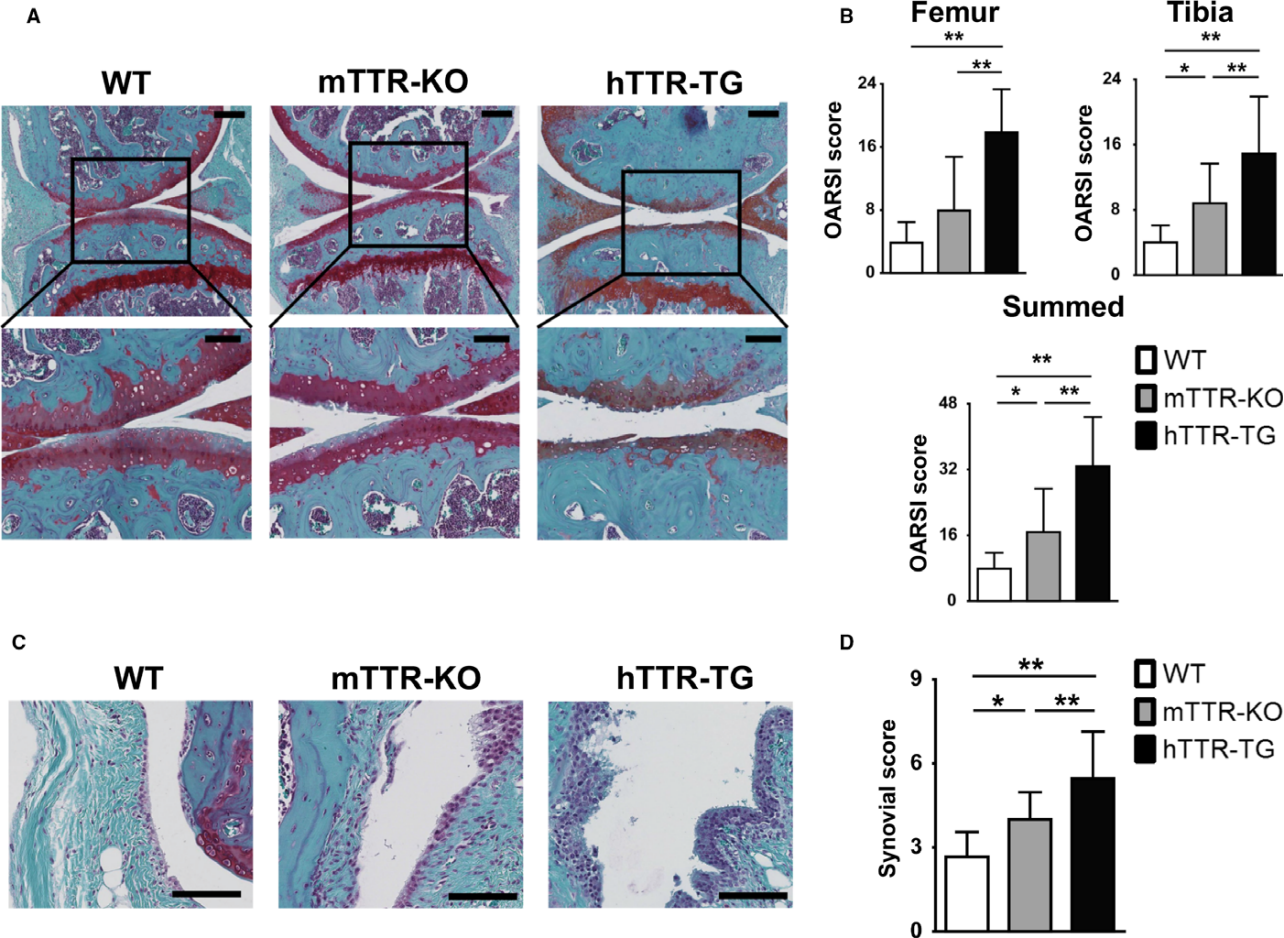
Cartilage and synovial lesions in surgically induced osteoarthritis (OA) in wild‐type (WT), mTTR‐KO, and hTTR‐TG mice. (A) Histological features of knee cartilage 10 weeks after OA surgery (safranin O/fast green; upper: 10×, scale bars = 200 μm, lower: 20×, scale bars = 100 μm). OA was surgically induced by destabilizing the medial meniscus in the right knee joints of 4‐month‐old male mice. (B) Histological OA grade was based on Osteoarthritis Research Society International (OARSI) scoring system. Cartilage scores were significantly higher in hTTR‐TG mice (*n* = 26) 10 weeks after surgery than in WT (*n* = 20) and mTTR‐KO mice (*n* = 22). (C) Histological changes in synovium 10 weeks after OA surgery (scale bars = 100 μm). (D) The synovial score was obtained with Krenn's synovitis scoring system. Synovial scores were significantly higher in hTTR‐TG mice (*n* = 26) 10 weeks after surgery than in WT (*n* = 20) and mTTR‐KO mice (*n* = 22). **P < 0.01 and *P < 0.05.

To investigate the effect of hTTR deposition on cartilage, we used the experimental OA model induced by surgical destabilization of the medial meniscus (DMM) in 4‐month‐old male WT, hTTR‐TG, and mTTR‐KO mice. Ten weeks after DMM surgery, cartilage scores were significantly higher in hTTR‐TG mice (*n* = 26) than in mTTR‐KO mice (*n* = 22) and WT mice (*n* = 20) (Fig. 2A,B). Synovial scores were also significantly higher in hTTR‐TG mice than in mTTR‐KO or WT mice (Fig. 2C,D). Surprisingly, scores were also significantly higher in the mTTR‐KO compared to the WT mice but far lower than in the hTTR‐TG animals.

**Figure 3:**
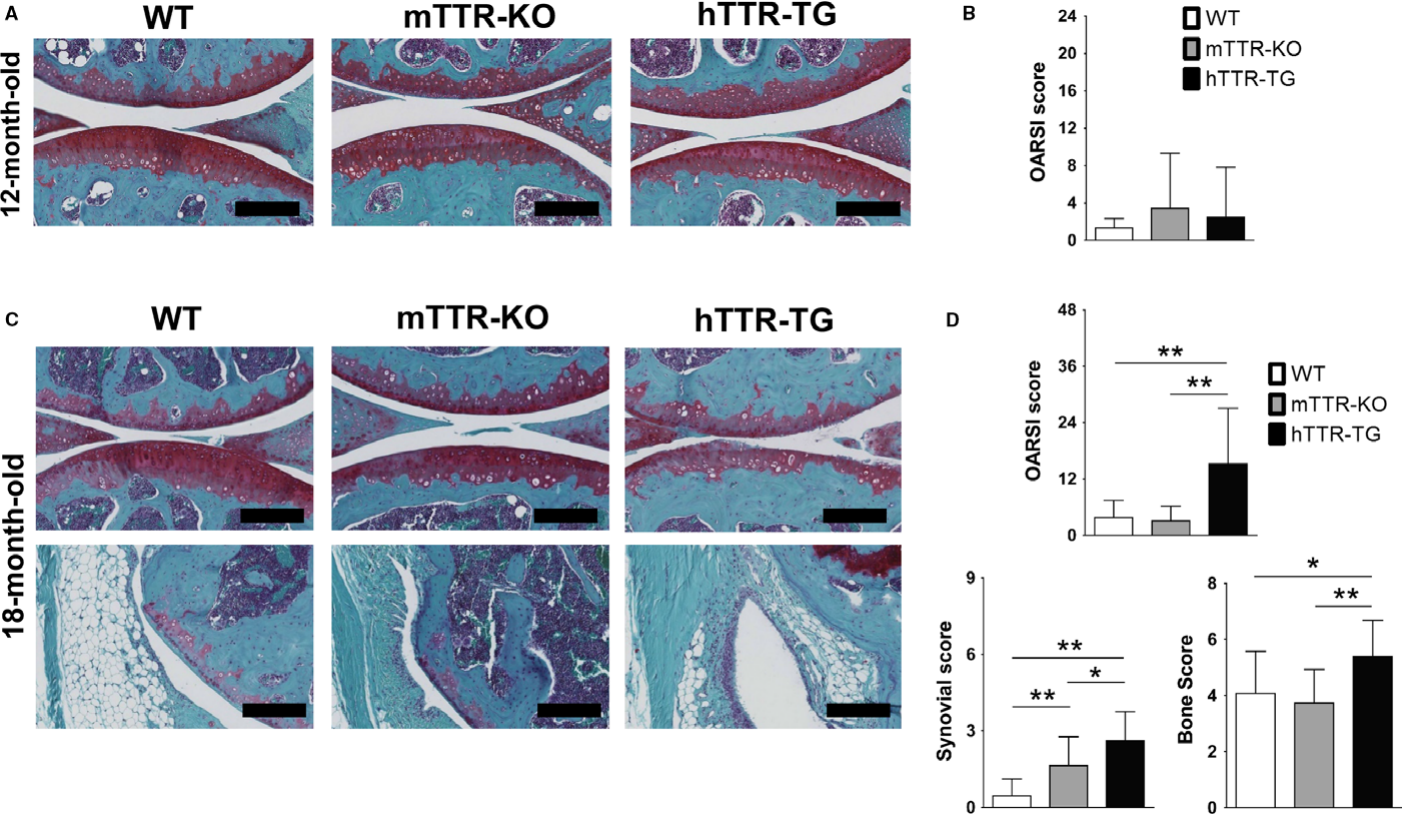
Cartilage, synovial, and subchondral bone histological changes in the aging model of wild‐type (WT), mTTR‐KO, and hTTR‐TG mice. (A) Histological features of cartilage in 12‐month‐old mice (safranin O/fast green; 20×, scale bars = 200 μm). (B) Histological osteoarthritis (OA) grades were based on OARSI scoring system. Cartilage scores were not significantly between WT (*n* = 12), mTTR‐KO mice (*n* = 14), and hTTR‐TG mice (*n* = 14) at 12 months of age. (C) Histological features of cartilage and synovium in 18‐month‐old mice (scale bars = 200 μm). (D) The synovial scores were obtained with Krenn's synovitis scoring system. Cartilage scores were significantly higher in hTTR‐TG mice (*n* = 18) than in WT (*n* = 13) and mTTR‐KO mice (*n* = 22) at 18 months of age. Synovial scores and subchondral bone scores were significantly higher in hTTR‐TG than in WT and mTTR‐KO mice. **P < 0.01 and *P < 0.05.

In the absence of surgical meniscus destabilization, hTTR‐TG mice showed increased severity of age‐related cartilage changes, synovitis, and subchondral bone changes at 18 months, which were not seen at 12 months (Fig. 3).

### Markers and mediators of OA pathogenesis

To further examine the mechanism underlying accelerated cartilage damage in the hTTR‐TG mice, markers and mediators of cartilage degradation were examined. Cartilage cell numbers were significantly lower in hTTR‐TG mice at 6 months of age and 10 weeks after DMM surgery as compared to WT and mTTR‐KO mice (Fig. S3A,B).

**Figure 4:**
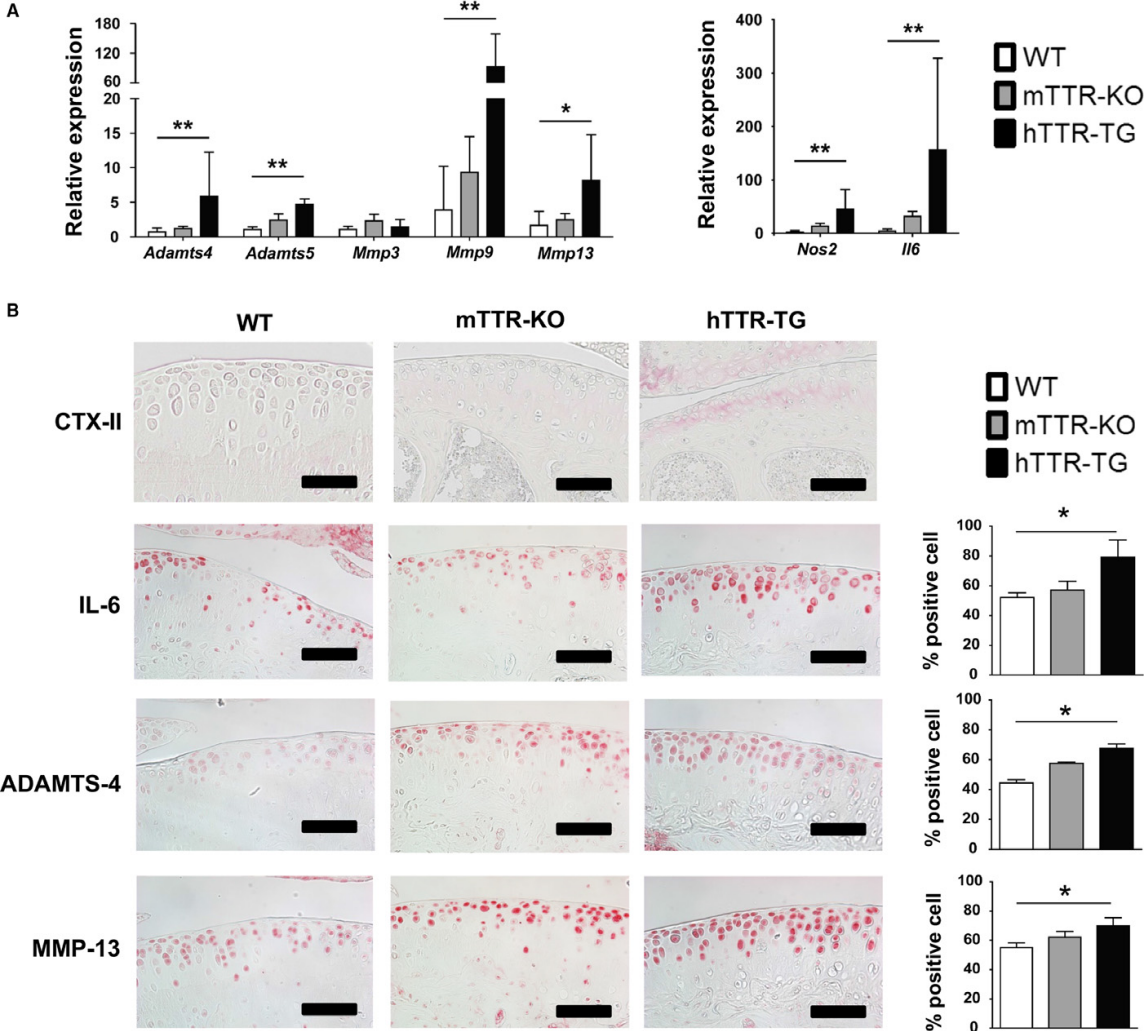
Mediators and markers of osteoarthritis (OA) in wild‐type (WT), mTTR‐KO, and hTTR‐TG mice. (A) RNA was isolated from knee articular cartilage from 6‐month‐old mice for RT‐qPCR. OA‐related genes were significantly elevated in cartilage from TTR‐TG mice (WT *n* = 6, mTTR‐KO *n* = 6, hTTR‐TG *n* = 5). The expression values were relative to *Gapdh*. **P < 0.01 and *P < 0.05. (B) Immunohistochemistry for CTX‐II, IL‐6, ADAMTS‐4, and MMP13 was performed on joint sections from 6‐month‐old mice (WT *n* = 4; mTTR‐KO *n* = 4; hTTR‐TG *n* = 4). Cells positive for IL‐6, ADAMTS‐4, and MMP13 were significantly elevated in 6‐month‐old hTTR‐TG mice (scale bars = 100 μm). The ratio of positive cells was counted using three sections from four mice for each genotype. *P < 0.05.

**Figure 5:**
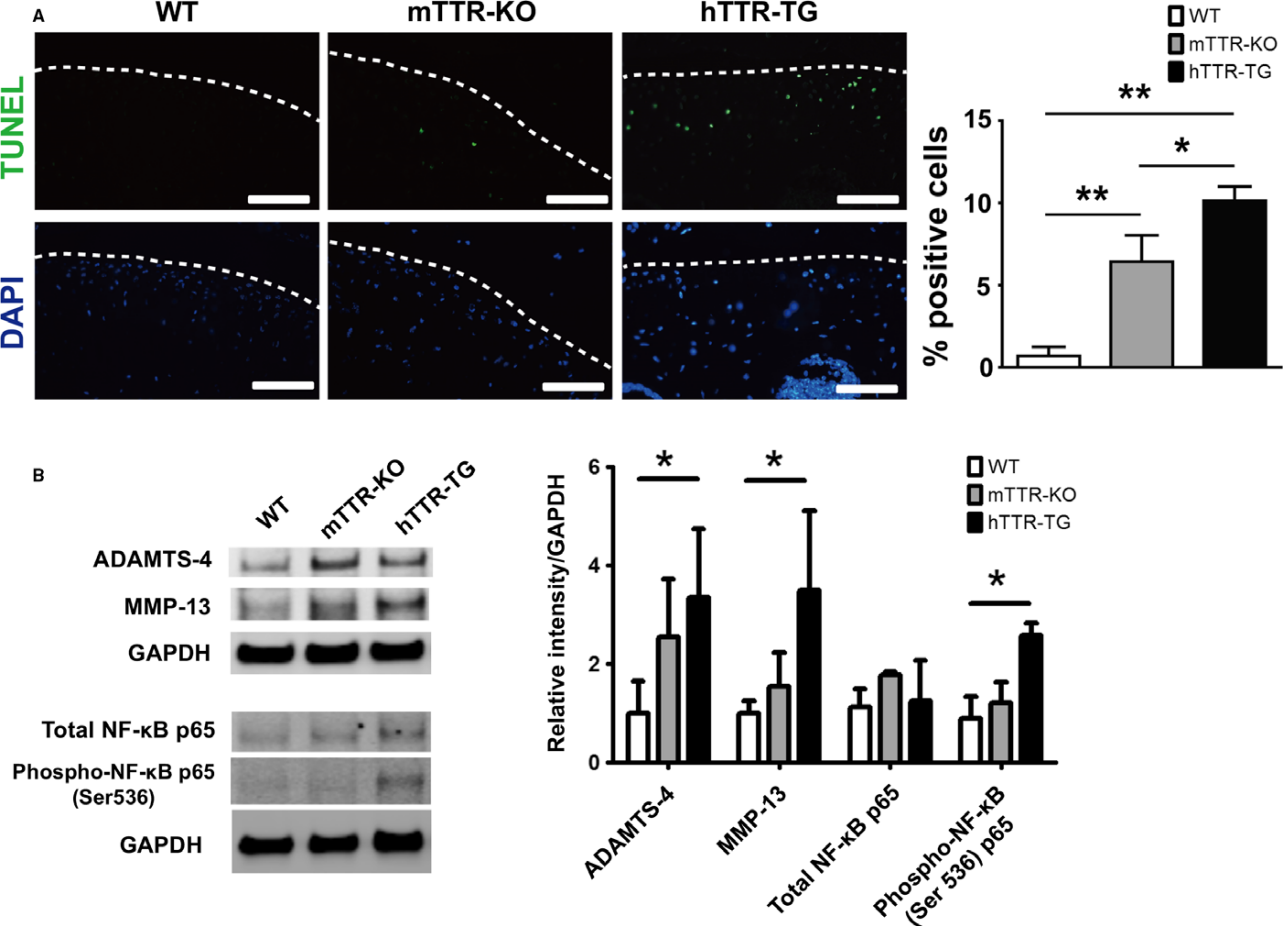
Apoptotic cells, MMP‐13, ADAMTS‐4 protein expression, and NF‐κB activation in cartilage from in WT, mTTR‐KO, and hTTR‐TG mice. (A) TUNEL staining (scale bars = 100 μm) and quantitative analysis of the TUNEL‐positive cell counts per field in the central weight‐bearing region of the medial tibial plateau in 12‐month‐old mice (*n* = 4, each). White dot shows the surface of cartilage. **P < 0.01. (B) Western blotting was performed on protein extracts from cartilage in 6‐month‐old WT, mTTR‐KO, and hTTR‐TG mice. Quantitative analysis of band intensity was measured using Image Studio (LI‐COR, USA). GAPDH served as a control. ADAMTS‐4, MMP‐13, and NF‐κB p65 were significantly elevated in hTTR‐TG mice (*n* = 4, each). *P < 0.05.

Real‐time PCR of mRNA from knee cartilage revealed that transcription of the catabolic genes *Adamts‐4 and 5*,* Mmp9*,* Mmp13*,* Nos2*, and the cytokine *Il‐6* was significantly elevated in 6‐month‐old hTTR‐TG mice compared to mTTR‐KO and WT mice (Fig. 4A). Immunohistochemistry showed higher intensity staining for the cartilage degradation marker CTX‐II in 6‐month‐old hTTR‐TG mice, and the number of cells positive for IL‐6, ADAMTS‐4, and MMP13 was significantly increased in 6‐month‐old hTTR‐TG mice (Fig. 4B). In addition, synovium of 6‐month‐old hTTR‐TG mice stained more intensely with antibodies for IL‐6, ADAMTS‐4, and MMP13 (Fig. S4). Furthermore, TUNEL staining showed that apoptotic cells in 12‐month‐old hTTR‐TG and mTTR‐KO mice were increased as compared to WT mice (Fig. 5A). Consistent with immunohistochemistry, in Western blots, the levels of ADAMTS‐4 and MMP‐13 were higher in hTTR‐TG mice (Fig. 5B).

The NF‐κB pathway regulates the production of inflammatory mediators and cartilage matrix‐degrading enzymes (Lauder *et al*., 2007). We found that in the 6‐month‐old hTTR‐TG mice, the protein level of NF‐κB p65 phosphorylated at serine 536 was significantly increased compared with control mice (Fig. 5B).

### Intra‐articular injection of TTR in WT mice

**Figure 6:**
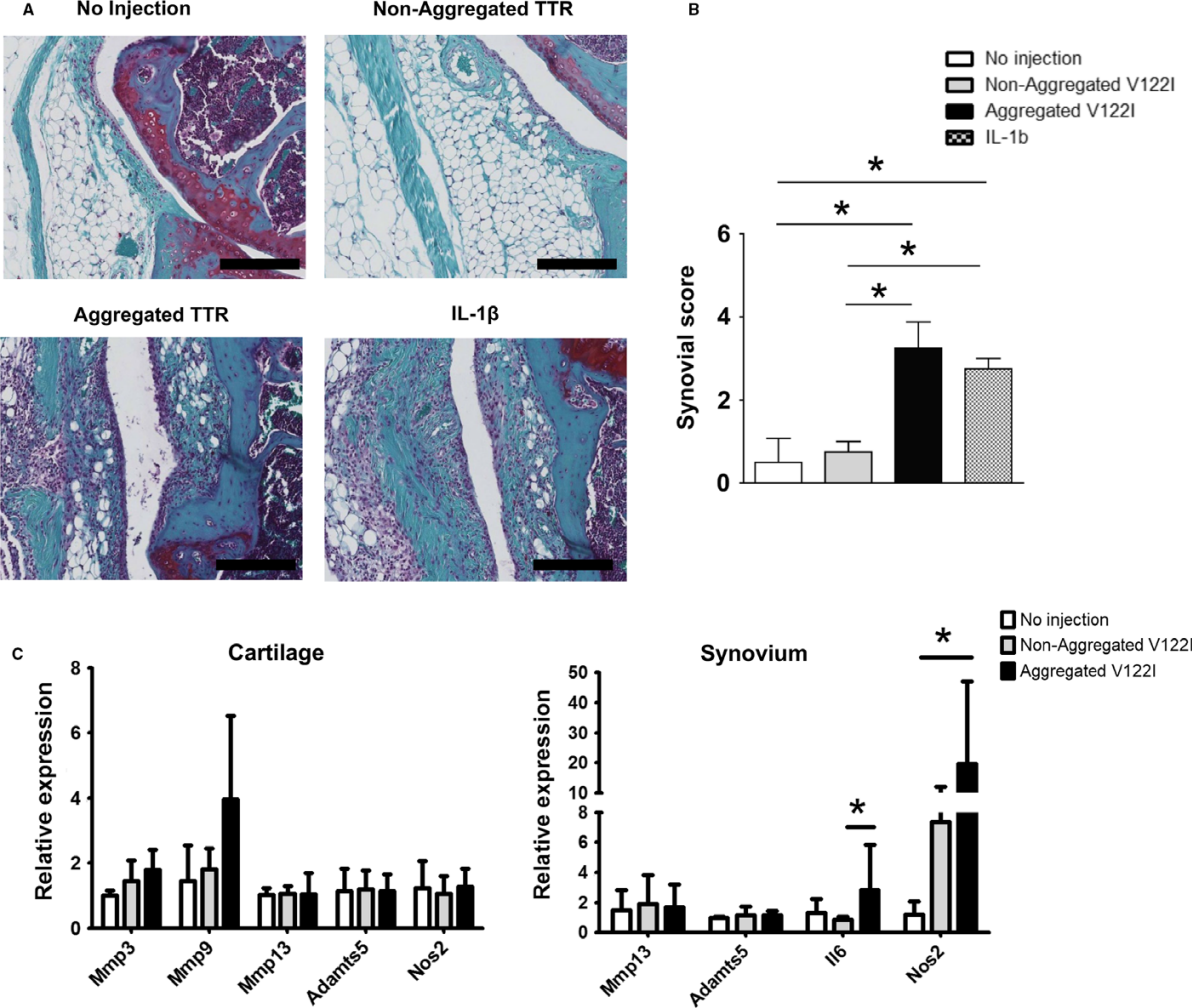
intra articular injection of aggregated transthyretin (TTR) in wild‐type (WT) mice. (A) Histological changes in synovium of 4‐month‐old WT mice 5 days after intra‐articular injections of aggregated TTR, nonaggregated V122 l TTR, or IL‐1β (scale bars = 200 μm). (B) Synovial scores were significantly higher in joints injected with aggregated V122I TTR and IL‐1β than nonaggregated V122I TTR or joints not receiving injections (each *n* = 4). *P < 0.05. (C) RNA was isolated from knee articular cartilage 1 week after injection. Inflammatory genes were significantly elevated in synovium from joints injected with aggregated V122I TTR (*n* = 5) than in joints injected with nonaggregated V122I TTR (*n* = 6) or not injected (*n* = 4). The values of mRNA expression were relative to *Gapdh*. *P < 0.05.

To examine the effect of aggregated TTR on the knee joint, intra‐articular injections of TTR were performed in WT mice. Injection of aggregated V122I led to increased synovitis (Fig. 6A,B) in a similar magnitude as IL‐1β, which was included as a positive control for a stimulus that is known to induce catabolic and inflammatory mediators in joint tissues (van de Loo *et al*. 1995). Real‐time PCR of mRNA from synovium also showed that inflammatory genes *Nos2* and *IL‐6* were significantly elevated in mice injected with aggregated TTR V122I compared to mice without injection or injection of nonaggregated TTR (Fig. 6C).

## Discussion

The deposition of amyloid in aged and OA‐affected cartilage has been documented in several publications (Goffin, 1983; Ladefoged, 1986; Solomon *et al*., 2006; Gobezie *et al*., 2007; Niggemeyer *et al*., 2011) with TTR being the predominantly identified amyloid precursor (Goffin, 1983). The consequences of the presence of amyloid deposits for tissue integrity and OA pathogenesis have not been explored. In this study, we used transgenic mice overexpressing wild‐type human TTR and mTTR‐KO mice to study the role of TTR deposition in joint tissues. We used mice with transgenic overexpression of human rather than mouse TTR as the mouse protein is kinetically several orders of magnitude more stable than the human protein and hence is not subject to amyloid formation, which depends on tetramer dissociation (Reixach *et al*., 2008).

TTR deposition was not detected in the cartilage of 6‐ and 12‐month‐old hTTR‐TG mice, but 69% of 18‐month‐old hTTR‐TG mice were positive for TTR deposits in cartilage. All hTTR‐TG mice were positive for TTR immunostaining in cartilage in the surgically induced OA model. However, Congo red staining for amyloid fibrils was negative in all mice tested. Hence, the TTR deposits were nonfibrillar.

The hTTR‐TG mouse strain that we have studied showed nonfibrillar, noncongophilic anti‐human TTR‐positive deposits between 12 and 17 months of age in the kidneys and heart (Teng *et al*., 2001). In mice over 18 months of age, TTR‐related deposits were found in 84% in the kidneys and 39% in the heart, while Congo red staining was found in 17% of the kidneys and in 16% of the hearts (Teng *et al*., 2001). Congophilic deposition was rarely seen before 18 months of age (Teng *et al*., 2001). The difference between the mice showing cardiac deposition and those that do not is whether their livers mount an unfolded protein response (UPR) (Buxbaum *et al*., 2008) and the nature of the inflammatory response in the heart. As those responses consist of endogenous mouse chaperones and inflammatory molecules, they do not appear to be related to the species specificity of the UPR and other chaperones involved in the organismal response to overexpression of human TTR in mice.

Our previous study on human cartilage revealed Congo red‐positive amyloid deposits in 58% of aged normal cartilage and all OA cartilage samples (Akasaki *et al*., 2015). TTR was detected in all OA cartilage samples and 83% of aged normal cartilage samples by immunohistochemistry (Akasaki *et al*., 2015). Collectively studies on wild‐type and mutant TTR indicate that age‐related changes in the tissue environment and/or the precursor protein are likely to be required for the deposition of TTR aggregates. The finding that TTR deposits formed in cartilage from young hTTR mice with surgically induced OA suggests that OA‐related cartilage changes promote TTR deposition, which, in turn, seems to amplify the OA damage.

We examined potential sources of TTR. In WT mice, the levels of TTR mRNA are very low in cartilage as compared to liver, the main source of TTR production (Holmgren *et al*., 1991, 1993). The level of hTTR mRNA in cartilage was also very low in the hTTR‐TG mice. The hTTR transgene in these mice contains all known regulatory regions responsible for tissue‐specific TTR expression (Teng *et al*., 2001) and thus recapitulates the physiological tissue distribution of TTR. We reported previously that TTR levels in human synovial fluid were similar to plasma, and there were no differences between OA and normal (Akasaki *et al*., 2015). Collectively, the prior and current data from the mouse models suggest that it is not a change in TTR production, but changes in the tissue environment and possible posttranslational changes in TTR that are the likely drivers of TTR deposition in arthritic joints.

The main observation from the present study is that hTTR‐TG mice develop more severe cartilage damage and synovitis than WT and mTTR‐KO mice in the surgically induced OA model and aging model. Although these mice were negative for amyloid fibrils as detected by Congo red staining, the observation is consistent with recent evidence from studies on tissues with TTR deposits indicating that inflammation, apoptosis, and reactive oxygen species are present in human carriers of mutant TTR variants and transgenic mice, well before amyloid deposits can be detected (Sousa *et al*., 2002; Starck & Sutherland‐Smith, 2010). In addition, that we were using, the hearts of 3‐month‐old transgenic mice overexpressing human WT TTR had a significant increase in the transcription levels of genes related to inflammation and immune response, compared to nontransgenic littermates (Buxbaum *et al*., 2012) even though these transgenic animals do not display cardiac TTR deposition until 18 months of age (Buxbaum *et al*., 2012). The hTTR‐TG mice did not present abnormalities in skeletal development and there were no differences in joint pathology compared to WT and mTTR‐KO mice by 12 months. However, at 18 months, hTTR‐TG mice developed significantly increased OA degeneration and synovial changes compared to WT and mTTR‐KO mice.

One of the main mechanisms of TTR amyloid pathogenesis is cytotoxicity (Reixach *et al*., 2004; Akasaki *et al*., 2015). We showed that amyloidogenic TTR‐induced cell death in cultured chondrocytes (Akasaki *et al*., 2015) and one of the histological features of the hTTR mice was reduced cartilage cellularity in the DMM model. Thus, it appears that this is also one factor contributing to the increased OA severity in these mice.

It has been demonstrated that TTR activates the receptor for glycation end products (RAGE) and stimulates nuclear translocation of NF‐κB (Sousa *et al*., 2000). Consistent with those observations, we have previously found that an anti‐RAGE antibody inhibited V122I TTR variant‐induced chondrocyte iNOS gene expression (Akasaki *et al*., 2015). It is known that RAGE is increased in OA cartilage, apparently mediating the induction of metalloproteinases and inflammatory mediators through the NF‐κB pathway (Loeser *et al*., 2005; Steenvoorden *et al*., 2006). The consequences of that activation are increased cartilage degradation, synovitis, and chondrocyte hypertrophy (Cecil *et al*., 2005; Steenvoorden *et al*., 2006). Further, our data indicate the cartilage‐degrading enzymes ADAMTS‐4 and MMP‐13, and the cytokine IL‐6 were all increased in the 6‐month‐old hTTR‐TG mice. In addition, NF‐κB p65 phosphorylation was increased in hTTR‐TG mice compared with control mice, suggesting that the increase in inflammatory mediators in hTTR‐TG mice might be related to activation of the NF‐κB pathway. To directly test effects of TTR on joint tissues, we performed intra‐articular injections in WT mice and found that aggregated but not nonaggregated TTR led to synovitis and increased expression of inflammatory genes in synovium. These mice were only observed for 5 days after the TTR injections to establish proof‐of‐concept that TTR aggregates are biologically active *in vivo*. Longer term injections would be necessary to observe changes in articular cartilage.

An additional mechanism of TTR and inflammation relates to the formation of autoantibodies against TTR, which have been reported in rheumatoid arthritis (Sharma *et al*. 2014) and juvenile idiopathic arthritis (Clement *et al*. 2016). Misfolding, aggregation, and oxidation of TTR enhanced its immunogenicity in a transgenic mouse model (Clement *et al*. 2016) and other posttranslational modifications of TTR were observed in human RA sera (Ni *et al*. 2013). However, there was no difference in autoantibodies against TTR between OA patients and healthy controls (Sharma *et al*. 2014).

In addition to TTR deposition in cartilage and synovium, it is also possible that TTR aggregates may form in subchondral bone marrow in OA‐affected joints and contribute to changes in subchondral bone and cartilage. The close association of changes in subchondral bone and articular cartilage has been recognized in human OA and in animal models (Weinans *et al*. 2012). Furthermore, systemic increases in inflammatory mediators due to TTR deposition in extraarticular tissues such as liver and serum may contribute to increased joint inflammation and OA severity.

The present study also revealed, somewhat paradoxically, that mice with deletion of the TTR gene had significantly increased OA severity as compared to wild‐type mice in OA model. This implies a previously unknown physiological function of TTR that appears to be required for joint homeostasis independent of the amyloidogenicity of human TTR. Murine TTR is 80% homologous to human TTR. Nonetheless, it is not amyloidogenic because of its greater kinetic stability (Reixach *et al*., 2008). Our study shows that skeletal development and growth are not significantly different between WT mice and mTTR‐KO mice, even though there are reduced levels of serum retinol, retinol‐binding protein, and thyroid hormone (Palha *et al*., 1994). However, our results suggest that there may be effects of TTR deficiency on articular cartilage that may result in increased susceptibility to OA in the setting of induced joint damage. The role of thyroid hormones in regulating hypertrophic chondrocyte differentiation is well known (Williams, 2013), and recent studies indicate that thyroxin for which TTR functions as a carrier protein stimulated cartilage formation in mesenchymal stem cells (Lee *et al*., 2015). However, only limited information is available on the role of thyroxine in articular chondrocytes (Rosenthal *et al*., 2003). Further analyses are needed to elucidate mechanisms of TTR and thyroxin in articular cartilage homeostasis.

Our findings that TTR amyloid deposition is highly prevalent in aging and OA‐affected joints (Akasaki *et al*., 2015) and that TTR promotes inflammation and cartilage in mouse models suggest that TTR deposition contributes to the progression of OA. Recent advances in the understanding of mechanisms of TTR aggregation (Eisele *et al*., 2015) led to the discovery of TTR stabilizers such as tafamidis or diflunisal (Adamski‐Werner *et al*., 2004; Obici & Merlini, 2014) and tafamidis have been approved for the treatment of familial amyloidotic polyneuropathy caused by amyloid formed by mutant TTR (Coelho *et al*., 2013; Waddington Cruz & Benson, 2015). Evaluation of these drugs in preclinical models of OA will be the next step in establishing proof‐of‐concept that prevention of TTR aggregation is a potential therapeutic approach for OA.

This study is the first to show that transgenic overexpression of TTR led to deposition on cartilage and increased the severity of cartilage damage and synovitis in the surgically induced murine OA model. Together with prior observations that aging and OA in humans are associated with TTR and amyloid deposition in cartilage, the present findings suggest that reducing TTR amyloid formation can be a new therapeutic approach for OA.

## Experimental procedures

### Mice

hTTR‐transgenic (hTTR‐TG) and mice lacking endogenous *Ttr* genes (mTTR‐KO) mice were generated as described previously (Teng *et al*., 2001), and wild‐type mice of the same Sv129 strain (WT) were used. All procedures were performed according to protocols approved by the Institutional Animal Care and Use Committee (IACUC) at The Scripps Research Institute. All mice were freely allowed to access to food, water, and activity.

### Cell culture and Western blotting analysis

Chondrocytes were isolated from the femoral condyle and tibial plateau of postnatal day 1 control and hTTR‐TG mice (*n* = 5, pooled), as described previously (Gosset *et al*., 2008). Chondrocytes were seeded on 6‐well plates (2 × 10^5^/well) and incubated for 24 h, and protein was extracted as described (Akasaki *et al*., 2015). Proteins were separated by NuPAGE (Thermo Fisher Scientific, Waltham, MA, USA) and transferred to nitrocellulose. The following antibodies were used: human TTR (Dako, Santa Clara, CA, USA), ADAMTS‐4 (Abcam, Cambridge, MA, USA), MMP‐13 (Abcam), nuclear factor‐κB (NF‐κB) p65 (Cell Signaling, Danvers, MA, USA), and phospho‐NF‐κB p65 (Ser536) (Cell Signaling). The primary antibodies were added and incubated at 4°C overnight. After secondary antibody application, signal detection was performed using an LI‐COR immunofluorescence detection system as described (Akasaki *et al*., 2014).

### Surgical OA model and aging model in mice

To examine the effect of TTR overexpression on induced cartilage damage, we analyzed hTTR‐TG mice and compared them with WT and mTTR‐KO mice using an experimental OA model (Glasson *et al*., 2007). OA was surgically induced by destabilizing the medial meniscus (DMM) in the right knee joints of 4‐month‐old male WT, mTTR‐KO, and hTTR‐TG mice. The left knees were subjected to sham surgery. The mice were killed at 10 weeks after surgery, and the entire knee joints were fixed in 10% zinc‐buffered formalin for 2 days and decalcified in TBD‐2 for 24 h. Sections of the mouse knee joints were used for further analysis. The destabilized and sham‐operated joints were processed and analyzed in parallel. To examine the role of hTTR during aging, we compared WT, mTTR‐KO, and hTTR‐TG mice at 12 and 18 months.

### Intra‐articular injection of TTR

To examine the direct effect of aggregated TTR on knee joint tissue, intra‐articular injections of aggregated (25 μM), nonaggregated V122 l (25 μM), and IL‐1β (100 ng) were performed in 4‐month‐old WT mice three times on days 0, 2, and 4. Aggregated V122I solution was created using acetate buffer (200 mM sodium acetate/100 mM KCl/1 mM EDTA, pH 4.2) as described previously (Reixach *et al*., 2008) and incubated at 37°C for 3 days. The precipitate was diluted in Hank's balanced salt solution. One day after final intra‐articular injection, knee joints were collected for histological analysis and RNA isolation.

### Immunohistochemistry

Joint sections were deparaffinized, and the slides were washed and blocked with 10% goat serum for 1 h at room temperature. Antigen retrieval was used as necessary. Anti‐human TTR (Dako A0002; 1:400), anti‐mouse and human TTR (Abcam ab9015; 1:100), anti‐CTX‐II (Cloud‐Clone Corp. PAA686Hu01; 1:2000 dilution), anti‐ADAMTS‐4 (Abcam ab28285; 1:100 dilution), anti‐MMP‐13 (Acam ab39012; 1:100 dilution), anti‐IL‐6 (Abcam ab6672; 1:600 dilution), and negative control rabbit IgG (Vector I‐1000; 1 μg/mL) were applied with 0.1% Tween 20 and incubated overnight at 4°C, and followed by secondary antibody using Vectastain ABC‐AP alkaline phosphatase (Vector Laboratories, Burlingame, CA, USA). Slides were washed, and sections were incubated with alkaline phosphatase substrate for 5–10 min. Specificity controls were obtained by replacing the primary antibody with nonimmune rabbit IgG (1 μg/mL), and no staining was observed under these conditions. Hematoxylin was used for counter staining. The percent positive cells was determined as the ratio of the total number of positive cells to the total number of chondrocytes in the section. TUNEL staining was performed using *In Situ* Cell Death Detection Kit (Roche Applied Science, Indianapolis, IN, USA). The number of positive cells was quantified in tibial cartilage and the percent positive cells was determined as the ratio of the total number of TUNEL‐positive cells to the total number of DAPI‐positive cells in the section.

### RNA and protein isolation, and real‐time PCR

Articular cartilage was collected from both sides of the femoral head, femoral condyle, and tibial plateau of each mouse at the ages indicated as described previously (Gepstein *et al*., 2002; Xu *et al*., 2005). Care was taken to avoid any other tissue such as synovium in the cartilage collection and to resect full thickness articular cartilage without subchondral bone. In intra‐articular injection study, only knee joints were collected for RNA. Synovium was carefully resected and total RNA was extracted using TRIzol (Invitrogen, Burlington, ON), followed by Zymo Direct‐zol RNA MiniPrep kits (Zymo Research, Irvine, CA, USA), according to the manufacturer's instructions. Real‐time PCR was performed on a Light Cycler 480 instrument (Roche Diagnostics, Indianapolis, IN, USA) using the following TaqMan probes: MMP‐3 (Mm00440295_m1), MMP‐9 (Mm00442991_m1), MMP‐13 (Mm01168713_m1), ADAMTS‐4 (Mm00556068_m1), ADAMTS‐5 (Mm01344182_m1), NOS2 (Mm01309897_m1), IL‐6 (Mm00446190_m1). Protein isolation was performed on the residue from the RNA extraction (Akasaki *et al*., 2014).

### Histological analysis

The histological OA scores for medial femoral condyle, the medial tibial plateau, and summed scores of femur and tibia were evaluated using the Osteoarthritis Research Society International (OARSI) cartilage OA histopathology semiquantitative scoring system (score 0–24) (Pritzker *et al*., 2006). The histological synovial score was evaluated using Krenn's synovitis scoring system (score 0–9) (Krenn *et al*., 2002). To evaluate the subchondral bone, we used a scoring system (score 0–8) based on previous publications (Botter *et al*., 2011; Ko *et al*., 2013) that evaluates trabecular bone structure (mostly cystic: 2, partially cystic: 1), lamellar structure (destroyed: 2, partially destroyed: 1), angiogenesis (number of vessels ≥3: 2, 1–2: 1), and ectopic ossification/articular cartilage absorption in the tibia. (present: 2). Histological grading was performed by two blinded observers.

### Skeletal preparation

Whole skeletons of WT mTTR‐KO and hTTR‐TG mice on postnatal day 2 were fixed in 95% ethanol. Alcian blue staining was performed, followed by placement into potassium hydroxide, and alizarin red staining.

### Statistical methods

Descriptive statistics are summarized as mean ± standard deviation (SD). An unpaired two‐tailed Student's *t*‐test or Mann–Whitney *U*‐test was used to compare differences between two groups. One‐way analysis of variance was used to compare multiple groups, with subsequent pairwise (group) comparisons assessed via Bonferroni's procedure, at an experiment‐wise error level of 0.05. *P* values less than 0.05 were considered statistically significant.

## Funding

This study was supported by National Institutes of Health grant AG007996.

## Conflict of interest

The authors declare that they have no competing for financial interests.

## Author contributions

MKL had full access to all the data in the study and took responsibility for the integrity of the data and the accuracy of the data analysis. TM, JB, NR, and MKL designed research. TM, MO, YA, OA performed research. TM and MKL analyzed data. All authors involved in writing the paper.

## Supporting information


**Fig. S1** Whole mouse skeletal preparations. Double‐staining with alizarin red and alcian blue of whole skeletal preparations was performed on P2 WT, mTTR‐KO, and hTTR‐TG mice (scale bars = 500 μm). (B) Length of femur and tibia (scale bars = 2 mm) in P2 control (*n* = 7), mTTR‐KO (*n* = 7), and hTTR‐TG (*n* = 6) mice. N.S. = Non‐Significant.

**Fig. S2** TTR mRNA and protein expression in chondrocytes, cartilage, synovium, serum, and liver. (A) Chondrocytes were isolated from the femoral condyle and tibial plateau of postnatal day 1 (P1). Cartilage, liver and serum protein were extracted from 9‐month‐old mice. Human recombinant TTR was used as a positive control. Western blotting showed that TTR was detected in cartilage in 9‐month‐old hTTR‐TG mice, but expressed at low levels in chondrocytes. High TTR expression was observed in liver and serum in hTTR‐TG but not in mTTR‐KO. (B) Real‐time PCR for human *Ttr* in 2‐month‐old hTTR‐TG mice showed lower expression in cartilage than liver (*n* = 3, each). (C) Real‐time PCR for mouse *Ttr* in 2‐month‐old wild‐type C57BL/6 mice showed that cartilage and synovium were lower in primary chondrocytes (*n* = 6).

**Fig. S3** Cartilage cellularity. (A) Representative images medial tibia cartilage of Safranin O stained joint sections. B) Three micrographs of the medial tibial plateau were taken under 40× magnification. Cell numbers per each area were counted in 6‐month‐old mice and mice with surgical OA (scale bars = 400 μm, 6 months: WT *n* = 20, hTTR‐TG *n* = 28, mTTR‐KO *n* = 28, Surgical model: WT *n* = 20, hTTR‐TG *n* = 26, mTTR‐KO *n* = 22). **P < 0.01.

**Fig. S4** Expression of OA‐related markers in synovium. Immunohistochemistry for IL‐6, ADAMTS‐4, and MMP13 was performed on joint sections from 6‐month‐old mice (WT *n* = 4; mTTR‐KO *n* = 4; hTTR‐TG *n* = 4, scale bars = 100 μm).

**Fig. S5** Immunohistochemistry with antibody that recognizes human and mouse TTR. (A) TTR was detected in bone marrow, blood vessels and at the articular cartilage surface of 6‐month‐old hTTR‐TG and WT mice but not in mTTR‐KO mice (scale bars = 400 μm). (B) TTR was detected in fibrillated cartilage in 18‐month‐old hTTR‐TG mice and in chondrocyte in hTTR‐TG and WT mice. There was no TTR staining in the cartilage in 18‐month‐old mTTR‐KO mice (scale bars = 100 μm).
