# Supplementary figures and images for: Transthyretin deposition promotes progression of osteoarthritis
Source: Aging Cell. 2017 Sep 22;16(6):1313–22. doi: 10.1111/acel.12665 (PMC5676063; doi:10.1111/acel.12665)

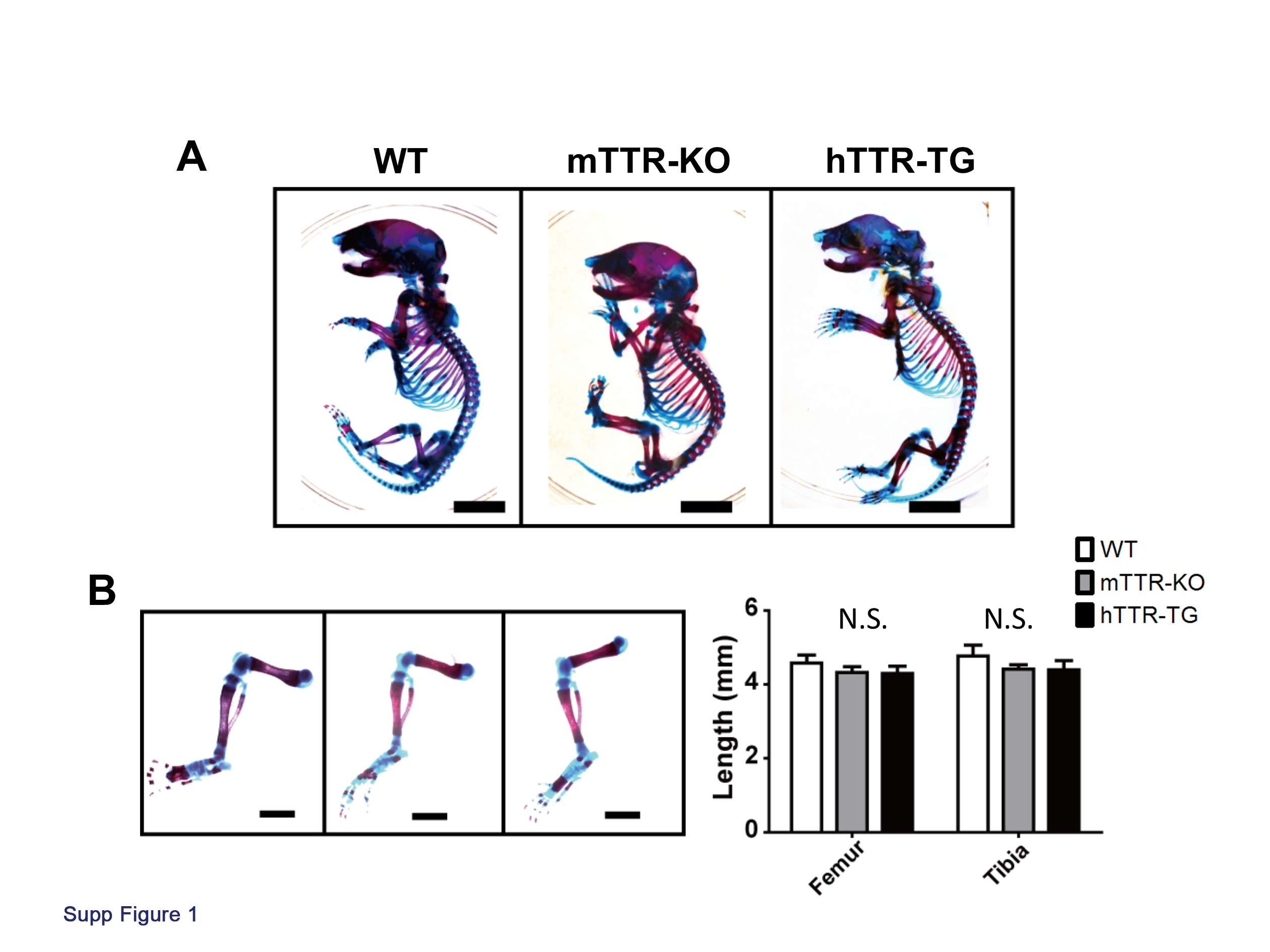

Supplement: Supplementary file 1 — Fig. S1 Whole mouse skeletal preparations. Double‐staining with alizarin red and alcian blue of whole skeletal preparations was performed on P2 WT, mTTR‐KO, and hTTR‐TG mice (scale bars = 500 μm). (B) Length of femur and tibia (scale bars = 2 mm) in P2 control (n = 7), mTTR‐KO (n = 7), and hTTR‐TG (n = 6) mice. N.S. = Non‐Significant. [file ACEL-16-1313-s001.tif]

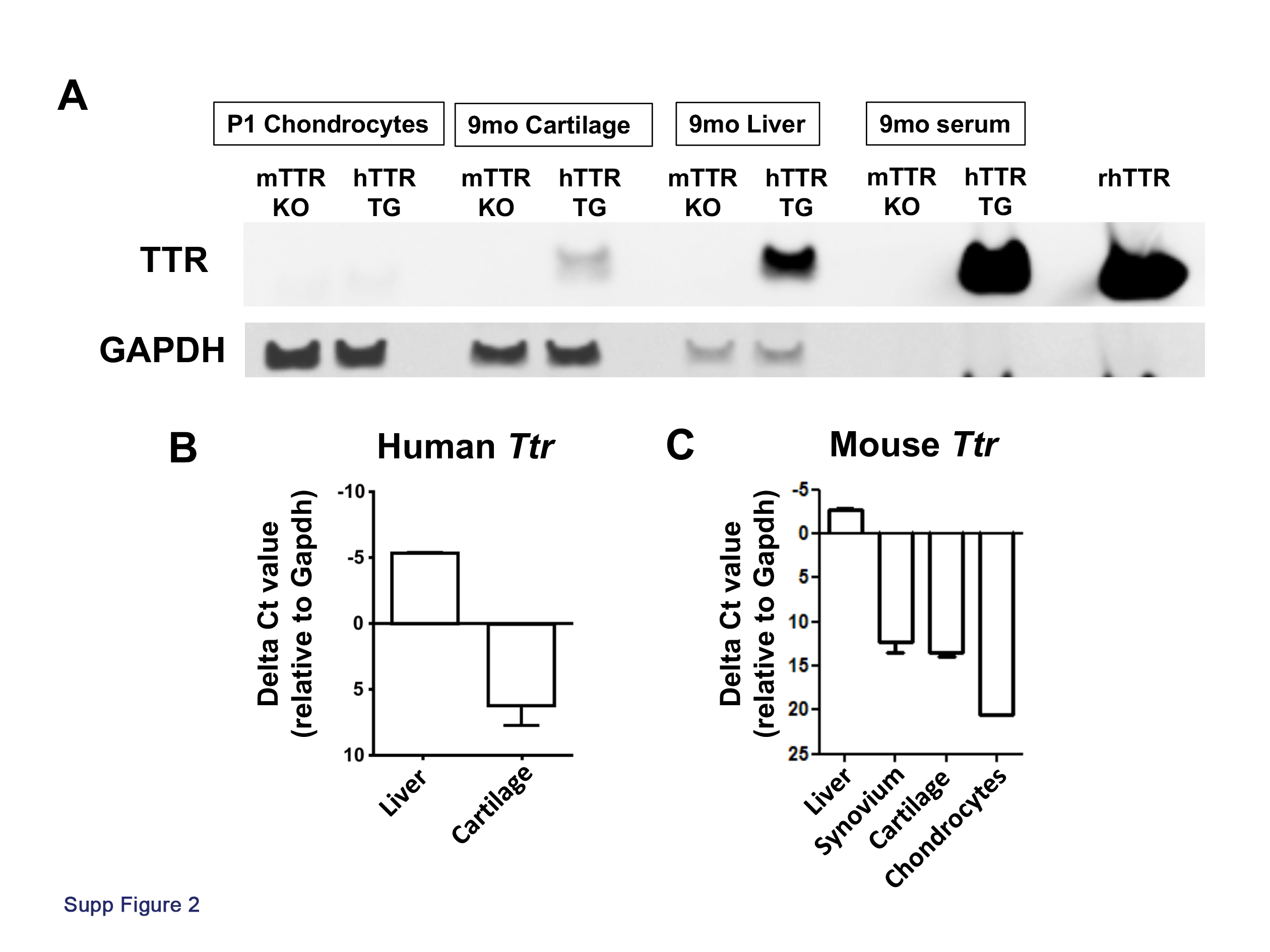

Supplement: Supplementary file 2 — Fig. S2 TTR mRNA and protein expression in chondrocytes, cartilage, synovium, serum, and liver. (A) Chondrocytes were isolated from the femoral condyle and tibial plateau of postnatal day 1 (P1). Cartilage, liver and serum protein were extracted from 9‐month‐old mice. Human recombinant TTR was used as a positive control. Western blotting showed that TTR was detected in cartilage in 9‐month‐old hTTR‐TG mice, but expressed at low levels in chondrocytes. High TTR expression was observed in liver and serum in hTTR‐TG but not in mTTR‐KO. (B) Real‐time PCR for human Ttr in 2‐month‐old hTTR‐TG mice showed lower expression in cartilage than liver (n = 3, each). (C) Real‐time PCR for mouse Ttr in 2‐month‐old wild‐type C57BL/6 mice showed that cartilage and synovium were lower in primary chondrocytes (n = 6). [file ACEL-16-1313-s002.tif]

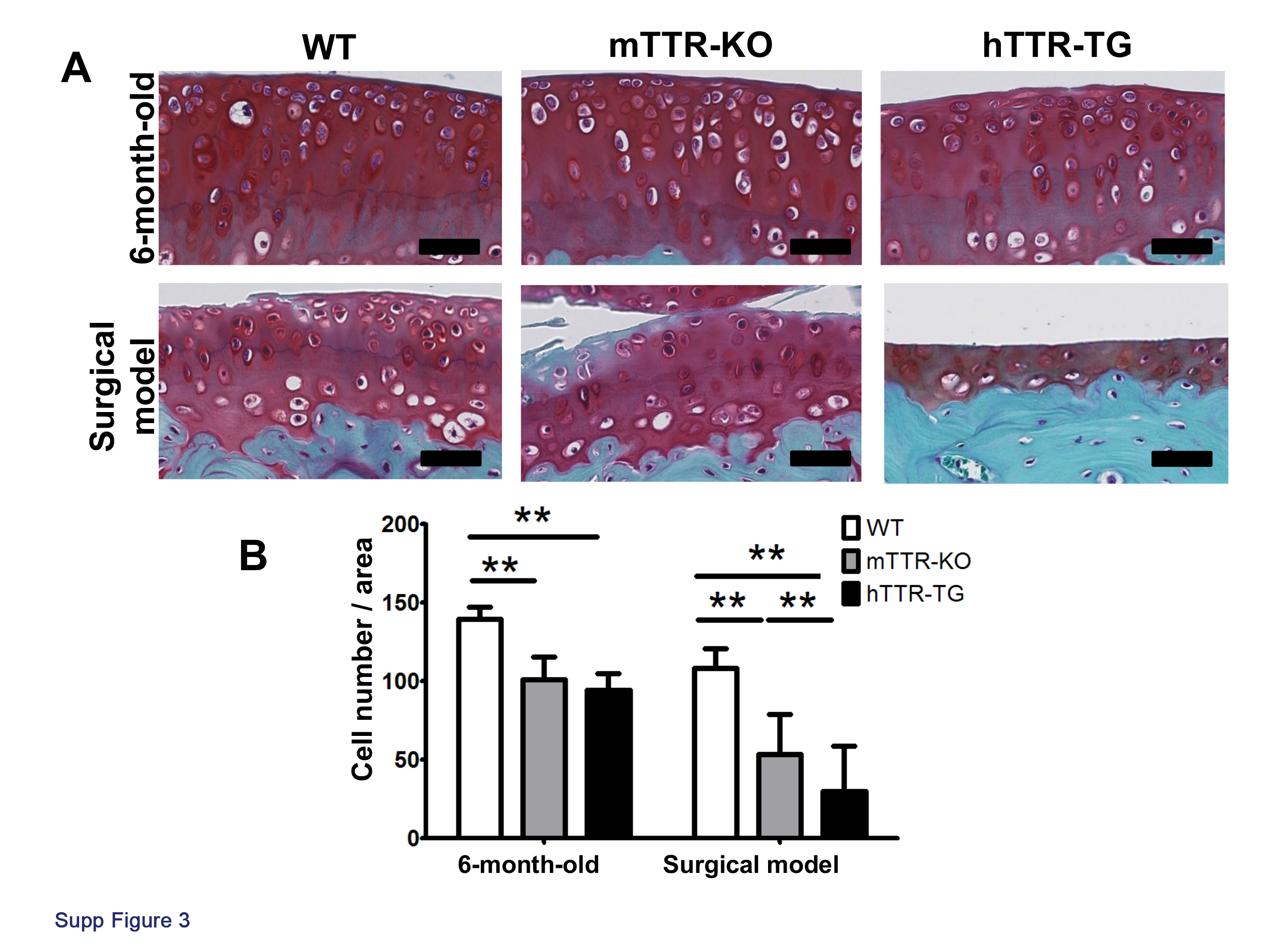

Supplement: Supplementary file 3 — Fig. S3 Cartilage cellularity. (A) Representative images medial tibia cartilage of Safranin O stained joint sections. B) Three micrographs of the medial tibial plateau were taken under 40× magnification. Cell numbers per each area were counted in 6‐month‐old mice and mice with surgical OA (scale bars = 400 μm, 6 months: WT n = 20, hTTR‐TG n = 28, mTTR‐KO n = 28, Surgical model: WT n = 20, hTTR‐TG n = 26, mTTR‐KO n = 22). **P < 0.01. [file ACEL-16-1313-s003.tif]

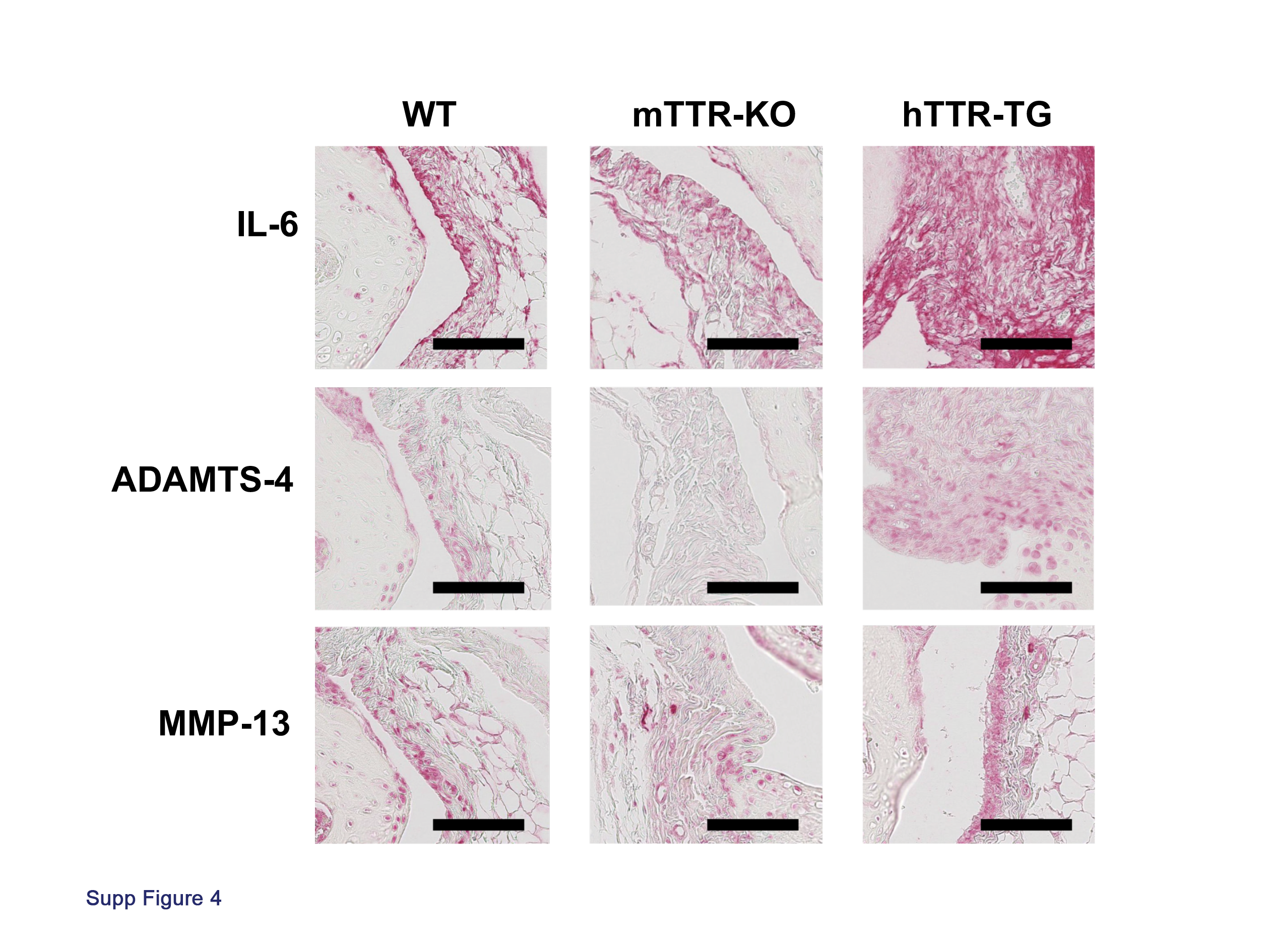

Supplement: Supplementary file 4 — Fig. S4 Expression of OA‐related markers in synovium. Immunohistochemistry for IL‐6, ADAMTS‐4, and MMP13 was performed on joint sections from 6‐month‐old mice (WT n = 4; mTTR‐KO n = 4; hTTR‐TG n = 4, scale bars = 100 μm). [file ACEL-16-1313-s004.tif]

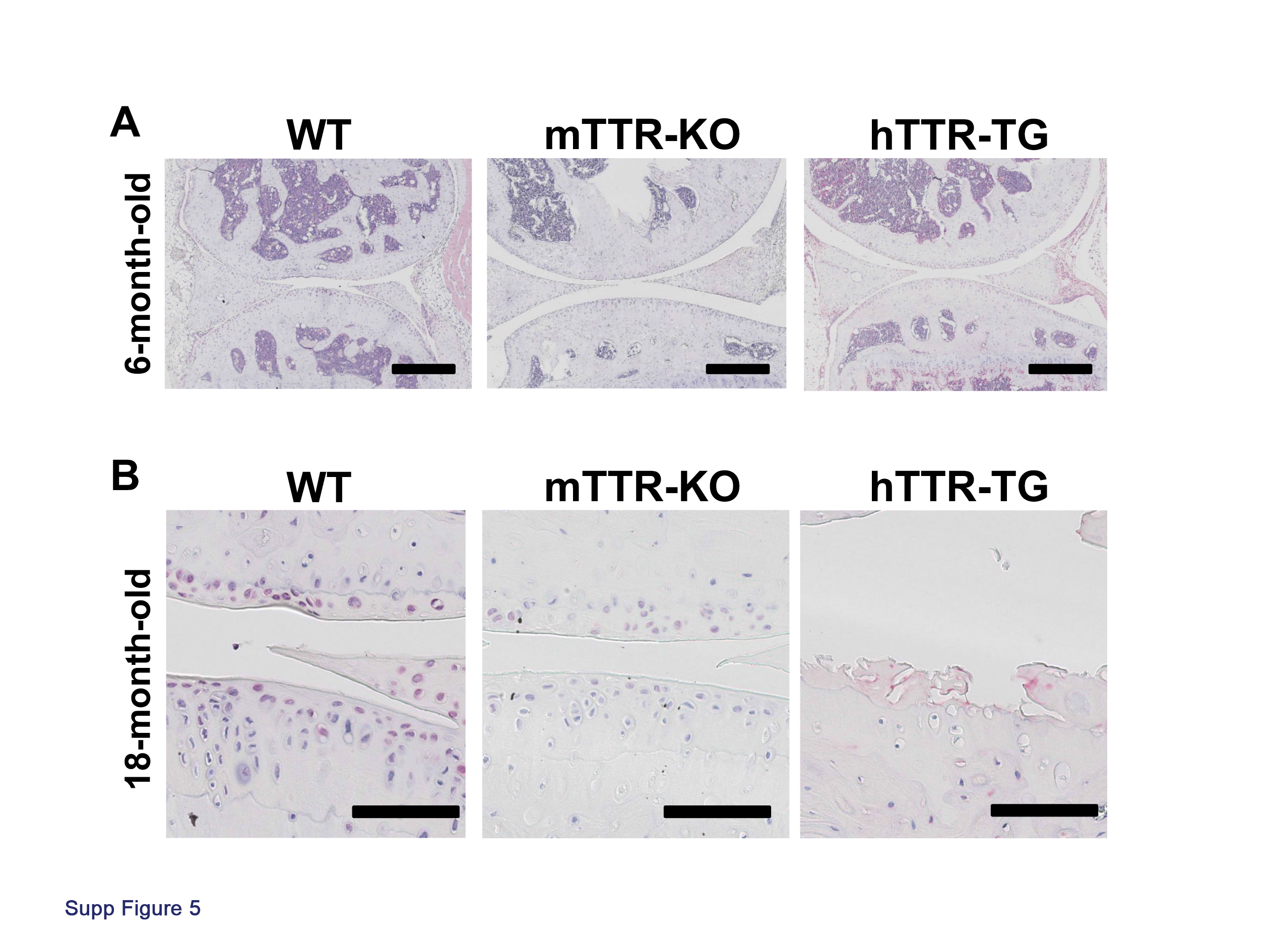

Supplement: Supplementary file 5 — Fig. S5 Immunohistochemistry with antibody that recognizes human and mouse TTR. (A) TTR was detected in bone marrow, blood vessels and at the articular cartilage surface of 6‐month‐old hTTR‐TG and WT mice but not in mTTR‐KO mice (scale bars = 400 μm). (B) TTR was detected in fibrillated cartilage in 18‐month‐old hTTR‐TG mice and in chondrocyte in hTTR‐TG and WT mice. There was no TTR staining in the cartilage in 18‐month‐old mTTR‐KO mice (scale bars = 100 μm). [file ACEL-16-1313-s005.tif]
